# Identifying Dietary Patterns Associated with Mild Cognitive Impairment in Older Korean Adults Using Reduced Rank Regression

**DOI:** 10.3390/ijerph15010100

**Published:** 2018-01-09

**Authors:** Dayeon Shin, Kyung Won Lee, Mi-Hye Kim, Hung Ju Kim, Yun Sook An, Hae-Kyung Chung

**Affiliations:** 1 Department of Public Health, Food Studies and Nutrition, Syracuse University, Syracuse, NY 13244, USA; dshin03@syr.edu; 2 Division of Epidemiology and Health Index, Center for Genome Science, Korea National Institute of Health, Korea Centers for Disease Control and Prevention, Cheongju-si 28160, Korea; kyungwlee@korea.kr; 3 Department of Food and Nutrition, Hoseo University, Asan, Chungnam 31499, Korea; kimmihye92@hoseo.edu; 4 Department of Social Welfare, Wonkwang University, Iksan 54538, Korea; kanddol@wku.ac.kr (H.J.K.); 200233sook@naver.com (Y.S.A.)

**Keywords:** cognitive function, dietary patterns, reduced rank regression (RRR), Korean-Mini Mental State Examination (K-MMSE), older Korean adults

## Abstract

Diet plays a crucial role in cognitive function. Few studies have examined the relationship between dietary patterns and cognitive functions of older adults in the Korean population. This study aimed to identify the effect of dietary patterns on the risk of mild cognitive impairment. A total of 239 participants, including 88 men and 151 women, aged 65 years and older were selected from health centers in the district of Seoul, Gyeonggi province, and Incheon, in Korea. Dietary patterns were determined using Reduced Rank Regression (RRR) methods with responses regarding vitamin B6, vitamin C, and iron intakes, based on both a one-day 24-h recall and a food frequency questionnaire. Cognitive function was assessed using the Korean-Mini Mental State Examination (K-MMSE). Multivariable logistic regression models were used to estimate the association between dietary pattern score and the risk of mild cognitive impairment. A total of 20 (8%) out of the 239 participants had mild cognitive impairment. Three dietary patterns were identified: seafood and vegetables, high meat, and bread, ham, and alcohol. Among the three dietary patterns, the older adult population who adhered to the seafood and vegetables pattern, characterized by high intake of seafood, vegetables, fruits, bread, snacks, soy products, beans, chicken, pork, ham, egg, and milk had a decreased risk of mild cognitive impairment compared to those who did not (adjusted odds ratios 0.06, 95% confidence interval 0.01–0.72) after controlling for gender, supplementation, education, history of dementia, physical activity, body mass index (BMI), and duration of sleep. The other two dietary patterns were not significantly associated with the risk of mild cognitive impairment. In conclusion, high consumption of fruits, vegetables, seafood, and protein foods was significantly associated with reduced mild cognitive impairment in older Korean adults. These results can contribute to the establishment of dietary guidelines targeting older Korean adults to reduce mild cognitive impairments. Future prospective cohort studies are warranted to examine the effect of the seafood and vegetable dietary pattern on reducing mild cognitive impairment to prove the cause–effect relationship between dietary patterns and cognitive function.

## 1. Introduction

The number of older adults has rapidly risen worldwide, and cognitive decline is one of the major issues in older adults, associated with adverse health effects such as an increased risk for mortality [1], depressive symptoms [2], and metabolic syndrome [3]. Korea is rapidly transitioning from an ageing society to elderly society. The older adult Korean population, aged 65 or older, with mild cognitive impairment was estimated at 28.5% in 2011 [4].

Dietary factors play a key role in the prevention of cognitive decline. Dietary pattern approaches have been introduced to examine the synergistic effects of foods and nutrients, rather than examining a single nutrient in isolation. Some studies have investigated dietary patterns associated with cognitive functions in older Asian adults, including Korean [5,6] and Japanese [7] populations. Using cluster analysis, two major dietary patterns were identified in an older Korean adult population. Among the two dietary patterns, a dietary pattern including consumption of multi-grain rice, fish, dairy products, fruits, and fruit juices was associated with a lower risk of cognitive impairment [6]. To extract dietary patterns in these studies, empirical data-driven approaches, including principal component analysis (PCA) and cluster analysis, were used to input food or nutrient groups as predictors. The reduced rank regression (RRR) approach was used to input both food groups and responses, such as biomarkers and nutrients, as predictors that are known to be associated with disease. RRR is an innovative method that can elucidate the diet-disease pathway compared to classic PCA [8]. RRR has been used in relation to the risk of numerous diseases such as gestational diabetes mellitus [9], type 2 diabetes mellitus [10], depressive symptoms [11], and esophageal cancer [12]. In a study in Japan, the relationship between dietary patterns identified by RRR and the risk of dementia was examined [7]. Because Korea has distinct and traditional dietary patterns and cultures [13] compared to other Asia or western countries, identifying specific dietary patterns of the older Korean adult population is required.

To the best of our knowledge, few studies have identified the dietary patterns of the older adult Korean population using RRR techniques in relation to the degree of cognitive function. The aim of the study was to identify distinct dietary patterns in relation to cognitive impairment using RRR. We hypothesized that dietary patterns derived by RRR are differentially associated with the degree of cognitive function in the adult Korean population.

## 2. Materials and Methods

### 2.1. Study Design and Participants

A sample of 316 older adults aged ≥65 years who reside either in the district of Seoul, Gyeonggi province, or Incheon in Korea, were recruited through a one-to-one interview with questionnaires from March 2012 to July 2012. Of the 316 participants, 239 participants, including 88 men and 151 women, completed both a 24-h recall and a food frequency questionnaire (FFQ). The survey was conducted in the University Hospital clinics, the Elderly Welfare Centers, and the Health Welfare Center in the district of Seoul, Gyeonggi province, and Incheon in Korea. The study was reviewed and approved by the Institutional Review Board at Hannam University (2012-01K).

### 2.2. Identification of Dietary Patterns

Dietary intake data of older adults were collected by using both a one-day 24-h dietary recall and a 63-item FFQ designed to assess food and nutrient intakes among Koreans. The FFQ included 63 food and beverage items with 9 intake frequency and 4 serving size questions. The modified version of the FFQ from the Korea National Health and Nutrition Examination Survey (KNHANES) was previously validated in reference to 3-day diet records with correlation coefficients ranging from 0.3 to 0.7 [14]. Participants were asked to choose from 9 possible frequency responses, ranging from “never” to “3 times a day” for each food or beverage item. The intake was generated by multiplying the frequency of each food or beverage item by portion size of that food or beverage on a daily basis. Intakes of total energy, proteins, fats, carbohydrates, vitamins, and minerals were calculated from the one-day 24-h dietary recall data. In addition to completing FFQ, only one day 24-h recall was conducted to minimize the burden on respondents as they are older adults. A total of 63 food items were aggregated into 26 food groups. RRR was performed to extract dietary patterns of the older adult population with responses including intakes of vitamin C, vitamin B6, and iron. Responses chosen for RRR were nutrients that have been associated with cognitive impairment in an older adult population in the previous literature (vitamin B6, vitamin B12, vitamin C, vitamin E, calcium, zinc, folate, iron, and saturated fat). Analysis using different numbers of responses indicated that the greatest explanation of the total variation in food groups and in responses was obtained using vitamin B6, vitamin C, and iron (14.2% in food groups and 17.8% in responses). Results of this analysis is presented in Supplemental Table S1.

### 2.3. Assessment of Cognitive Function

Cognitive function was examined by the Korean-adjusted version of the Mini-Mental State Examination (K-MMSE), which is the most widely used screening tool for quantitative assessment of the cognitive status of the older adult population, developed by Kwon and Park [15], adapted from Folstein et al. [16]. The K-MMSE includes 19 items covering the following fields: orientation (10 points), language function (6 points), attention and calculation (5 points), memory (3 points), memory recall (3 points), understanding and judgment (2 point), and visuospatial construction (1 point). The scores range from 0 to 30. A higher K-MMSE score indicates better cognitive function. The study population with normal cognitive function were those who had a K-MMSE score equal or greater than −1.5 standard deviations (SD) of the mean score [17]. The study population with mild cognitive impairment were those who had a K-MMSE less than −1.5 SD of the overall mean MMSE score [17].

### 2.4. Covariates

The multivariate model was adjusted for sex, supplementation, education, history of dementia, physical activity, body mass index (BMI), and sleep duration. Sex was categorized as men or women. Supplementation was divided into yes or no. Education level was categorized as uneducated, ≤high school, and graduate college or higher. History of dementia was divided into yes or no. Physical activity was categorized as yes or no. BMI (kg/m^2^) and sleep duration (hours/day) were controlled in a continuous variable.

### 2.5. Statistical Analyses

Descriptive statistics were used to examine the frequencies and means (SD) of socioeconomic and lifestyle variables in relation to two categories of cognitive function status: mild cognitive function or normal cognitive function. Distributions of socioeconomic status, lifestyle variables, and nutrients were examined in relation to the tertiles of each dietary pattern. The chi-square test for categorical variables and the *t*-test for continuous variables were used to examine the differences in sociodemographic, lifestyle variables, and nutrients across the tertiles of the dietary pattern scores. Analysis of variance (ANOVA) was conducted to test if any significant differences existed across the tertiles of each dietary pattern score. Multivariable logistic regression was used to calculate adjusted odds ratios with 95% confidence intervals (CI) to examine the relationship between each dietary pattern and the risk of mild cognitive impairment after controlling covariates including sex, daily supplement use, education, history of dementia, physical activity, BMI, and daily sleep duration. All statistical analyses were performed using SAS (version 9.4; SAS Institute, Cary, NC, USA).

## 3. Results

Table 1 shows the differences in descriptive statistics of socioeconomic and lifestyle variables between older adults with mild cognitive impairment compared to those with normal cognitive functions. Sex, daily supplement use, education, self-reported health, tooth conditions, physical activity, age, BMI, and sleep duration significantly differ with cognitive function status (*p* value < 0.05).

Three dietary patterns were identified using RRR, as presented in Table 2, with factor loadings on each food or beverage group. The first dietary pattern called the “seafood and vegetables pattern” was characterized by a high intake of seafood, vegetables, bread, snacks, soy products, beans, chicken, pork, ham, egg, fruits, and milk. The second pattern, labeled “high meat pattern” was characterized by high consumption of whole grains, beef, and pork, and low consumption of soy products, sweet potatoes, eggs, and milk. The third pattern, named “bread, ham, and alcohol pattern” was characterized by high consumption of bread, ham, and alcohol, and low consumption of beans, sweet potatoes, pork, eggs, and seaweed.

**Figure 1:**
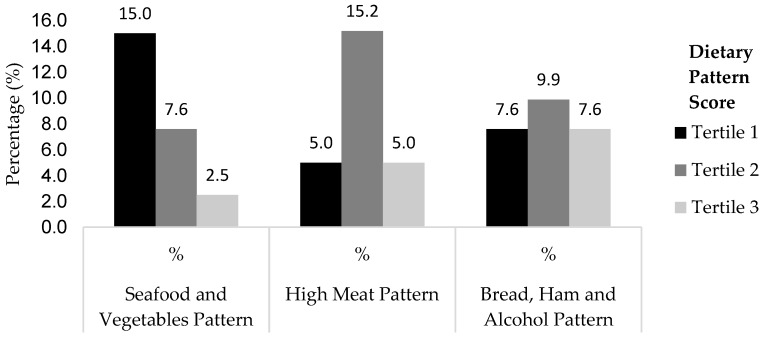
Prevalence of mild cognitive impairment across the tertiles of dietary pattern score.

The prevalence of mild cognitive impairment across the tertiles of the dietary pattern score is presented in Figure 1. The prevalence of older adults with mild cognitive impairment decreased with an increase in the seafood and vegetables pattern score, from the lowest tertile (15%) to the highest tertile (2.5%).

Socioeconomic status and lifestyle variables across the tertiles of each dietary pattern score is presented in Table 3. The distributions of daily supplement use, history of dementia, physical activity, age, and BMI significantly differed amongst the tertiles of the seafood and vegetables pattern (*p* value < 0.05). Self-reported health status, history of dementia, and K-MMSE significantly differed among the tertiles of the high meat pattern (*p* value < 0.05). Distributions of sex, family type, education, self-reported health status, smoking status, age, and K-MMSE significantly differed among the tertiles of the bread, am, and alcohol pattern (*p* value < 0.05).

Energy and nutrient intakes by the tertiles of each dietary pattern score are presented in Table 4. Protein as a percentage of energy, fiber, moisture, vitamin A, vitamin C, vitamin B2, niacin, vitamin B6, folate, calcium, phosphorous, sodium, potassium, iron, and zinc significantly increased as the seafood and vegetables pattern score increased from tertile one to tertile three (*p* value < 0.05). Vitamin C intake significantly increased from the lowest to the highest tertiles of the high meat pattern score (*p* value < 0.05). Intakes of fat as a percentage of energy, protein as a percentage of energy, saturated fatty acids, monounsaturated fatty acids, niacin, and vitamin B6 significantly increased, but folate and iron significantly decreased across the tertiles of the bread, ham, and alcohol pattern.

Adjusted odds ratios (AOR) and the 95% CI for mild cognitive impairment across the tertiles of each dietary pattern score are presented in Table 5. Older adults in the highest tertile of the seafood and vegetable pattern were less likely to have mild cognitive impairment compared to those older adults in the lowest tertile (AOR 0.06, 95% CI 0.01–0.72) after controlling for covariates (*p*-for-trend < 0.027). Each unit increase in the seafood and vegetable pattern score significantly lowered the odds for mild cognitive impairment (AOR 0.25, 95% CI 0.08–0.86). The number of older adults affected by mild cognitive impairment reduced from 12 in the lowest tertile of the seafood and vegetable pattern to two in the highest tertile of the seafood and vegetable pattern. The other two dietary patterns, high meat, and bread, ham, and alcohol, were not significantly associated with the risk of mild cognitive impairment.

## 4. Discussion

We identified three dietary patterns in the older Korean adult population aged 65 or older: seafood and vegetables, high meat, and bread, ham, and alcohol. Among the three patterns, the older adult population who adhered to the seafood and vegetables pattern had a decreased risk for mild cognitive impairment compared to those who did not. In the meantime, the other two dietary patterns were not associated with mild cognitive impairment. The very low value in explained variation for “High Meat” Pattern and “Bread, Ham, and Alcohol” Pattern could partially explain no significant association with cognitive impairment. Previous studies have identified specific dietary patterns related to the degree of cognitive function or Alzheimer’s disease using different dietary approaches, including factor analysis, cluster analysis, RRR, and index analysis. In a French older population [18], participants with the highest adherence to the healthy dietary pattern, characterized by high consumption of fruit, whole grains, fresh dairy products, and vegetables, demonstrated better cognitive functioning compared to those with the least adherence. In a prospective cohort study of U.S. older adults, Gu et al. [19] found that the older adults with higher intakes of salad dressing, nuts, fish, tomatoes, poultry, cruciferous vegetables, fruits, and dark and green leafy vegetables, and a lower intake of high-fat dairy products, red meat, organ meat, and butter, had a reduced risk of Alzheimer’s disease. In a cross-sectional study for older Chinese people [20], older adults with the vegetable-fruits pattern, characterized by high intakes of vegetables and fruits, soy products, and legumes, had a reduced risk of cognitive impairment. Overall, these previous studies indicated that the overlapping food groups that reduced the risk of cognitive impairment were fruits and vegetable containing high antioxidants that are associated with reduced risk of cognitive impairment [21].

In our study, the seafood and vegetable pattern indicating high consumption of fruits and vegetables lowering the risk of mild cognitive impairment is supported by a U.S. prospective cohort study [22] and a populations-based cohort of Australian adults [23]. In the Nurses’ Health Study [22], the effect of daily in fruit and vegetable intake on cognitive function and decline was examined in women aged 70 and older, and a higher vegetable intake was found to be associated with less cognitive decline. In a population of older adults aged 60 and older, the fruit and vegetable pattern, derived by PCA, was associated with a lower likelihood of cognitive impairment. High concentrations of antioxidants in fruits and vegetables demonstrated protective effects of cognitive decline in previous findings.

Older adults who adhered to the seafood and vegetables pattern had significantly higher intakes of protein as a percentage of energy, fiber, moisture, vitamin A, vitamin C, vitamin B2, niacin, vitamin B6, folate, calcium, phosphorous, potassium, iron, and zinc. Our findings align with previous findings. In a study of Italian older adults (mean age: 75.8 years), patients with mild cognitive impairment presented significantly lower plasma levels of vitamin C, vitamin E, and A compared to their counterparts [24]. Vitamin C is a powerful water soluble antioxidant that prevents the oxidative process by donating its electrons [25,26]. Another antioxidant, Vitamin E, has been found to have a beneficial role in reduced cognitive decline [27] by capturing reactive oxygen-free radicals and reducing oxidative stress [28]. Notably, a significantly higher intake of folate and vitamin B6 was found in those who followed the seafood and vegetable pattern compared to those who did not. Folate and vitamin B6 have been shown to have beneficial effects for cognitive decline. Folate deficiency is closely linked to elevated homocysteine, which has been associated with recall memory decline in aging men [29] and increased risk for cognitive impairment in an older adults in The Netherlands [30]. Folate is found in vegetables and may support the beneficial effect of the seafood and vegetables pattern on cognitive decline. Seafood consumption [31], tuna and dark-meat fish (one vs. less than one serving/week) has also been associated with lower decline in verbal memory during a four-year follow-up (OR 0.84, 95% CI 0.60–0.96) of women. In the present study, squid and clams contain high amounts of omega-3 fatty acids, including eicosapentaenoic acid (EPA) and docosahexaenoic acid (DHA), which have been to found to reduce amyloid-beta deposition, neuron loss, and eventually improve cognitive functioning as found by a meta-analysis [32].

Our study has several limitations. Firstly, due to the cross-sectional study design, the cause-effect relationship cannot be drawn in this study. Secondly, changes in cognitive functioning could not be captured in this study. However, dietary intake was assessed using both a food frequency questionnaire and 24-h recalls, which captured both short- and long-term dietary intake. Thirdly, although we controlled for a large number of covariates such as sex, supplemental use, education, history of dementia, physical activity, BMI, and sleep duration, we may have not accounted for residual confounding. Lastly, a single, one-day 24-h recall may not be representative of an individual’s usual diet.

## 5. Conclusions

In conclusion, the seafood and vegetables dietary pattern was significantly associated with reduced mild cognitive impairment in older Korean adults. These results may contribute to the establishment of dietary guidelines targeting older Korean adults to reduce mild cognitive impairment. The RRR is useful over other models since it reveals diet-disease pathway, and they are in line with global findings that may lead to the development of global recommendations. Future prospective cohort studies are warranted to examine the effects of the seafood and vegetable dietary pattern on reducing mild cognitive impairment to prove the cause–effect relationship between dietary patterns and cognitive function.

## Figures and Tables

**Table 1:** Sociodemographic characteristics of study participants between the degrees of cognitive function.

Sociodemographic Characteristics	K-MMSE				*p* Value ^1^
	Mild Cognitive Impairment (*n* = 20)		Normal (*n* = 219)		
	*n*	%	*n*	%	
Sex					
Men	2	10.0	86	39.3	0.009
Women	18	90.0	133	60.7	
Daily Supplements Use					
Yes	0	0.0	108	49.3	<0.0001
No	20	100.0	111	50.7	
Family Type					
With spouse	6	30.0	102	46.6	0.215
With children	7	35.0	75	34.3	
Spouse and children	1	5.0	15	6.9	
Alone	5	25.0	20	9.1	
Other	1	5.0	7	3.2	
Education					
Uneducated	15	75.0	36	16.4	<0.0001
≤High school Graduate	5	25.0	177	80.8	
College or Higher	0	0.0	6	2.7	
Self-Reported Health Status					
Very good	0	0.0	12	5.5	0.028
Good	6	30.0	42	19.2	
Fair	6	30.0	118	53.9	
Poor	4	20.0	36	16.4	
Very poor	4	20.0	11	5.0	
Self-Reported Teeth Condition					
Very good	2	10.0	10	83.3	<0.0001
Good	0	0.0	45	20.6	
Fair	2	10.0	78	35.6	
Poor	6	30.0	66	30.1	
Very poor	10	50.0	20	9.1	
History of Dementia					
Yes	2	10.0	10	4.6	0.287
No	18	90.0	209	95.4	
Smoking Status					
Yes	18	90.0	186	84.9	0.539
No	2	10.0	33	15.1	
Physical Activity					
None	12	60.0	56	25.6	<0.0001
1–2x/week	0	0.0	86	39.3	
3–4x/week	0	0.0	35	16.0	
Everyday	8	40.0	42	19.2	
**Variables**	**Mean**	**SD**	**Mean**	**SD**	***p* Value ^1^**
Age (years)	80.5	5.2	73.4	5.9	<0.0001
BMI (kg/m^2^)	18.3	4.2	22.1	3.5	<0.0001
Sleep Duration (hours/day)	5.2	2.7	6.9	1.6	0.0129

^1^ Chi-square test was used for categorical variables, and *t*-test was used for continuous variables. K-MMSE: Korean-Mini Mental State Examination; BMI: body mass index; SD: standard deviations.

**Table 2:** Factor loadings and explained variation in food groups, and response of dietary patterns from reduced rank regression (RRR) in an older Korean adult population.

Food Groups	Seafood and Vegetables Pattern	High Meat Pattern	Bread, Ham, and Alcohol Pattern
White rice	-	-	-
Whole grains	-	0.26	-
Noodles	-	-	-
Bread	0.21	-	0.34
Rice cakes	-	-	-
Snacks	0.25	-	-
Soy products	0.29	−0.39	-
Beans	0.29	-	−0.26
Potatoes	-	-	-
Sweet potatoes	-	−0.28	−0.29
Beef	-	0.32	-
Chicken	0.24	-	-
Pork	0.23	0.43	−0.24
Ham	0.22	-	0.31
Egg	0.27	−0.27	−0.44
Fish	-	-	-
Seafood (Fish cake, squid, clam, salted fish)	0.33	-	-
Vegetables	0.37	-	-
Seaweed	-	-	−0.26
Fruits	0.24	0.25	-
Milk	0.21	−0.36	-
Yogurt	-	-	-
Ice cream	-	-	-
Beverages	-	-	-
Alcohol	-	-	0.30
Fast foods	-	-	-
Explained variation in:			
Food groups (%)	5.30	3.67	3.06
Responses (%)	15.35	3.20	2.00

The following factors had loadings ≥|0.20| are shown in the table.

**Table 3:** Sociodemographics and lifestyles across the tertiles of each dietary pattern score.

Sociodemographic and Lifestyle Characteristics	Seafood and Vegetables Pattern							High Meat Pattern							Bread, Ham and Alcohol Pattern						
	Tertile 1 (*n* = 80)		Tertile 2 (*n* = 79)		Tertile 3 (*n* = 80)		*p* Value	Tertile 1 (*n* = 80)		Tertile 2 (*n* = 79)		Tertile 3 (*n* = 80)		*p* Value	Tertile 1 (*n* = 79)		Tertile 2 (*n* = 81)		Tertile 3 (*n* = 79)		*p* Value
	*n*	%	*n*	%	*n*	%		*n*	%	*n*	%	*n*	%		*n*	%	*n*	%	*n*	%	
Sex																					
Men	36	45.0	23	29.1	29	36.3	0.115	26	32.5	26	32.9	36	45.0	0.177	24	30.4	22	27.2	42	53.2	0.001
Women	44	55.0	56	70.9	51	63.8		54	67.5	53	67.1	44	55.0		55	69.6	59	72.8	37	46.8	
Supplements																					
Yes	28	35.0	36	45.6	44	55.0	0.039	41	51.3	29	36.7	38	47.5	0.161	41	51.9	28	51.9	39	49.4	0.058
No	52	65.0	43	54.4	36	45.0		39	48.8	50	63.3	42	52.5		38	48.1	53	65.4	40	50.6	
Family Type																					
With spouse	35	43.8	34	43.0	39	48.8	0.158	35	43.8	36	45.6	37	46.3	0.523	26	32.9	44	54.3	38	48.1	0.006
With children	26	32.5	28	35.4	28	35.0		26	32.5	24	30.4	32	40.0		35	44.3	24	29.6	23	29.1	
Spouse and children	4	5.0	3	3.8	9	11.3		6	7.5	8	10.1	2	2.5		3	3.8	2	2.5	11	13.9	
Alone	12	15.0	11	13.9	2	2.5		10	12.5	7	8.9	8	10.0		12	15.2	7	8.6	6	7.6	
Other	3	3.8	3	3.8	2	2.5		3	3.8	4	5.1	1	1.3		3	3.8	4	4.9	1	1.3	
Education																					
Uneducated	23	28.8	15	19.0	13	16.3	0.154	22	27.5	16	20.3	13	16.3	0.216	11	13.9	26	32.1	14	17.7	0.014
≤High school Graduate	57	71.3	61	77.2	64	80.0		57	71.3	62	78.5	63	78.8		68	86.1	52	64.2	62	78.5	
College or higher	0	0.0	3	3.8	3	3.8		1	1.3	1	1.3	4	5.0		0	0.0	3	3.7	3	3.8	
Self-Reported Health																					
Very good	4	5.0	4	5.1	4	5.0	0.792	1	1.3	2	2.5	9	11.3	0.005	9	11.4	0	0.0	3	3.8	0.019
Good	17	21.3	16	20.3	15	18.8		19	23.8	10	12.7	19	23.8		15	19.0	16	19.8	17	21.5	
Fair	41	51.3	45	57.0	38	47.5		39	48.8	46	58.2	39	48.8		38	48.1	40	49.4	46	58.2	
Poor	13	16.3	12	15.2	15	18.8		14	17.5	19	24.1	7	8.8		15	19.0	17	21.0	8	10.1	
Very poor	5	6.3	2	2.5	8	10.0		7	8.8	2	2.5	6	7.5		2	2.5	8	9.9	5	6.3	
Teeth Condition																					
Very good	5	6.3	0	0.0	7	8.8	0.144	4	5.0	5	6.3	3	3.8	0.132	7	8.9	2	2.5	3	3.8	0.197
Good	14	17.5	13	16.5	18	22.5		14	17.5	10	12.7	21	26.3		14	17.7	14	17.3	17	21.5	
Fair	23	28.8	29	36.7	28	35.0		27	33.8	23	29.1	30	37.5		27	34.2	21	25.9	32	40.5	
Poor	24	30.0	27	34.2	21	26.3		23	28.8	33	41.8	16	20.0		23	29.1	30	37.0	19	24.1	
Very poor	14	17.5	10	12.7	6	7.5		12	15.0	8	10.1	10	12.5		8	10.1	14	17.3	8	10.1	
History of Dementia																					
Yes	2	2.5	8	10.1	2	2.5	0.040	9	11.3	3	3.8	0	0.0	0.004	2	2.5	7	8.6	3	3.8	0.174
No	78	97.5	71	89.9	78	97.5		71	88.8	76	96.2	80	100.0		77	97.5	74	91.4	76	96.2	
Smoking																					
Yes	65	81.3	70	88.6	69	86.3	0.407	71	88.8	69	87.3	64	80.0	0.244	69	87.3	74	91.4	61	77.2	0.034
No	15	18.8	9	11.4	11	13.8		9	11.3	10	12.7	16	20.0		10	12.7	7	8.6	18	22.8	
Physical Activity																					
None	33	41.3	11	13.9	24	30.0	0.0007	15	18.8	30	38.0	23	28.8	0.099	19	24.1	25	30.9	24	30.4	0.288
1–2x/week	23	28.8	42	53.2	21	26.3		35	43.8	28	35.4	23	28.8		36	45.6	21	25.9	29	36.7	
3–4x/week	9	11.3	10	12.7	16	20.0		12	15.0	9	11.4	14	17.5		9	11.4	15	18.5	11	13.9	
Every day	15	18.8	16	20.3	19	23.8		18	22.5	12	15.2	20	25.0		15	19.0	20	24.7	15	19.0	
**Variables**	**Mean**	**SD**	**Mean**	**SD**	**Mean**	**SD**	***p* Value**	**Mean**	**SD**	**Mean**	**SD**	**Mean**	**SD**	***p* Value**	**Mean**	**SD**	**Mean**	**SD**	**Mean**	**SD**	***p* Value**
Age (years)	76.2	6.5	72.8	6.0	73.1	5.3	0.0004	75.0	6.7	73.9	4.9	73.2	6.5	0.155	72.9	5.7	75.8	6.6	73.4	5.8	0.005
BMI (kg/m^2^)	20.8	3.9	21.7	3.7	23.0	3.1	0.0004	21.4	3.3	22.1	4.3	22.0	3.4	0.411	22.1	3.2	21.3	4.1	22.1	3.7	0.254
Sleep Duration (hours/day)	7.1	1.9	6.5	1.6	6.6	1.8	0.067	6.6	1.9	7.0	1.8	6.6	1.6	0.233	6.8	1.7	6.7	1.9	6.7	1.8	0.886
MMSE	25.7	4.8	26.0	5.2	26.4	4.3	0.613	25.5	4.7	25.1	5.2	27.6	4.1	0.002	26.3	4.4	25.0	5.3	26.9	4.4	0.032

MMSE: mini mental state examination.

**Table 4:** Energy and nutrient intake across the tertiles of each dietary pattern.

Energy and Nutrient Intake Variables	Seafood and Vegetables Pattern							High Meat Pattern							Bread, Ham and Alcohol Pattern						
	Tertile 1		Tertile 2		Tertile 3		*p* Value	Tertile 1		Tertile 2		Tertile 3		*p* Value	Tertile 1		Tertile 2		Tertile 3		*p* Value
	Mean	SD	Mean	SD	Mean	SD		Mean	SD	Mean	SD	Mean	SD		Mean	SD	Mean	SD	Mean	SD	
Energy (kcal/day)	1519	273	1783	1668	1722	352	0.214	1613	286	1822	1672	1589	331	0.268	1608	283	1767	1651	1645	365	0.575
Carbohydrate (% of energy)	70.6	7.8	69.2	10.6	67.6	7.8	0.096	69.0	8.3	67.6	10.5	70.8	7.4	0.075	69.1	7.6	70.7	10.6	67.6	8.0	0.093
Fat (% of energy)	14.7	6.7	15.4	6.8	16.5	6.1	0.218	15.7	6.7	16.2	6.8	14.8	6.2	0.439	16.0	6.3	14.0	6.4	16.7	6.8	0.025
Protein (% of energy)	14.7	2.7	15.1	3.1	16.4	3.1	0.001	15.7	3.0	15.3	3.2	15.2	3.1	0.600	15.5	2.7	14.8	3.2	16.0	3.2	0.035
SFA (g/1000 kcal)	5.5	6.9	4.4	4.8	6.1	6.6	0.241	6.1	7.3	4.6	5.4	5.3	5.7	0.280	5.3	5.5	4.1	5.4	6.6	7.3	0.042
MUFA (g/1000 kcal)	7.2	9.3	6.0	6.7	7.9	8.8	0.334	8.0	10.1	5.9	7.1	7.2	7.5	0.268	7.2	7.5	5.4	7.2	8.6	9.9	0.050
PUFA (g/1000 kcal)	4.6	3.5	4.5	3.2	5.3	3.4	0.272	5.0	3.8	4.5	3.0	5.0	3.3	0.522	5.1	3.2	4.1	3.1	5.3	3.8	0.068
Fiber (g/1000 kcal)	13.7	2.9	14.7	3.5	15.1	3.7	0.029	14.3	3.7	14.4	3.6	14.9	3.0	0.458	14.9	3.2	14.2	3.4	14.3	3.6	0.370
Moisture (g/1000 kcal)	381.0	108.0	413.6	119.8	441.9	102.1	0.003	396.6	98.6	419.6	127.8	420.4	109.1	0.315	411.7	116.0	405.3	110.9	419.7	111.6	0.722
Vitamin A (μg/1000 kcal)	432.3	289.5	540.5	302.9	558.8	374.7	0.030	502.0	340.6	508.4	312.6	520.8	334.1	0.935	548.1	309.4	474.1	296.2	509.9	374.1	0.363
Vitamin E (mg/1000 kcal)	7.1	3.3	7.8	3.6	8.0	3.0	0.164	7.7	3.2	7.7	3.3	7.5	3.5	0.947	8.0	3.2	7.2	3.5	7.6	3.3	0.362
Vitamin C (mg/1000 kcal)	55.7	24.5	62.9	29.7	75.5	32.5	0.0001	58.4	24.4	63.8	31.1	71.9	33.0	0.016	68.1	32.4	63.5	29.6	62.5	28.2	0.458
Thiamin (mg/1000 kcal)	0.7	0.1	0.7	0.1	0.7	0.1	0.388	0.7	0.1	0.7	0.2	0.7	0.1	0.587	0.7	0.1	0.7	0.1	0.7	0.2	0.059
Vitamin B2 (mg/1000 kcal)	0.6	0.2	0.6	0.2	0.7	0.2	0.002	0.6	0.2	0.6	0.2	0.6	0.2	0.895	0.7	0.2	0.6	0.2	0.6	0.2	0.133
Niacin (mg/1000 kcal)	8.0	2.3	8.2	2.4	9.0	2.3	0.015	8.4	2.5	8.4	2.4	8.3	2.1	0.922	8.1	2.2	8.0	2.2	9.0	2.6	0.023
Vitamin B6 (mg/1000 kcal)	0.9	0.3	0.9	0.2	1.0	0.2	0.018	0.9	0.2	0.9	0.3	1.0	0.2	0.519	0.9	0.2	0.9	0.2	1.0	0.3	0.004
Folate (μg/1000 kcal)	337.8	91.4	359.8	118.3	387.6	131.7	0.025	370.0	137.3	338.9	95.9	376.1	109.8	0.097	389.4	136.5	350.8	110.2	345.4	95.2	0.034
Vitamin B12 (mg/1000 kcal)	4.8	5.9	6.6	7.4	6.3	4.9	0.127	6.7	6.7	5.2	4.5	5.8	7.0	0.320	6.3	6.5	5.8	7.0	5.6	4.8	0.756
Calcium (mg/1000 kcal)	294.3	108.6	314.6	106.6	347.9	116.3	0.009	329.6	110.6	315.9	109.3	311.3	117.5	0.566	333.7	113.9	309.7	107.1	313.7	115.9	0.353
Phosphorous (mg/1000 kcal)	610.9	138.1	626.5	143.0	663.5	125.7	0.044	633.3	131.3	630.7	143.6	636.9	137.7	0.960	628.0	126.6	623.4	144.1	649.8	139.8	0.432
Potassium (mg/1000 kcal)	1712.2	433.4	1757.4	440.3	1915.0	514.3	0.016	1829.8	524.9	1735.4	456.4	1819.0	424.5	0.385	1877.8	479.9	1718.8	425.9	1790.3	495.9	0.101
Iron (mg/1000 kcal)	8.2	1.7	8.9	2.1	9.7	2.8	<0.001	9.4	2.9	8.6	2.1	8.8	1.8	0.118	9.5	2.7	8.7	2.1	8.6	2.1	0.027
Zinc (mg/1000 kcal)	6.0	1.3	6.3	1.4	6.6	1.2	0.016	6.4	1.4	6.1	1.4	6.4	1.1	0.136	6.4	1.2	6.3	1.5	6.2	1.1	0.523
Copper (mg/1000 kcal)	0.8	0.2	0.8	0.2	0.8	0.2	0.530	0.8	0.2	0.8	0.3	0.8	0.2	0.698	0.8	0.2	0.8	0.2	0.8	0.2	0.597
Cholesterol (mg/1000 kcal)	145.1	103.8	135.0	90.1	128.9	73.9	0.515	143.5	102.1	134.6	84.8	130.9	82.3	0.666	144.4	97.5	129.2	87.8	135.7	84.6	0.565

**Table 5:** Adjusted odds ratios (AOR) for mild cognitive impairment across tertiles of each dietary pattern.

Dietary Patterns	Mild Cognitive Impairment (*n*)/Total (*n*)	AOR ^1^	95% CI
Seafood and Vegetables Pattern			
Tertile 1	12/80	1.00	
Tertile 2	6/79	0.45	0.04–5.63
Tertile 3	2/80	0.06	0.01–0.72
Continuous		0.25	0.08–0.86
*p*-for-trend		0.027	
High Meat Pattern			
Tertile 1	4/80	1.00	
Tertile 2	12/79	13.69	0.98–190.94
Tertile 3	4/80	1.25	0.06–25.90
Continuous		1.11	0.34–3.59
*p*-for-trend		0.866	
Bread, Ham and Alcohol Pattern			
Tertile 1	6/79	1.00	
Tertile 2	8/81	0.10	0.01–1.06
Tertile 3	6/79	0.56	0.08–3.83
Continuous		0.78	0.29–2.07
*p*-for-trend		0.611	

^1^ Adjusted for sex (men/women), supplemental use (yes/no), education (uneducated; ≤high school; graduate college or higher), history of dementia (yes/no), physical activity, BMI (kg/m^2^), and sleep duration (hours/day). AOR: adjusted odds ratios; 95% CI: 95% confidence intervals.

## Supplementary Materials

The following are available online at www.mdpi.com/1660-4601/15/1/100/s1, Table S1: Selection of responses variables in the reduced rank regression (RRR) analysis.
