# Supplementary material for: Identifying Dietary Patterns Associated with Mild Cognitive Impairment in Older Korean Adults Using Reduced Rank Regression
Source: Int J Environ Res Public Health. 2018 Jan 9;15(1):100. doi: 10.3390/ijerph15010100 (PMC5800199; doi:10.3390/ijerph15010100)
Supplement: Supplementary file 1 [file ijerph-15-00100-s001.pdf]

Supplemental

Table S1. Selection of responses variables in the reduced rank regression (RRR) analysis.

	**1st Model**	**2nd Model**	**3rd Model**	**4th Model**	**Final Model**
Responses	Vitamin B6	Vitamin B6	Vitamin B6	Vitamin B6	Vitamin B6
	Vitamin B12				
	Vitamin C	Vitamin C	Vitamin C	Vitamin C	Vitamin C
	Vitamin E	Vitamin E			
	Calcium	Calcium			
	Zinc	Zinc	Zinc		
	Folate	Folate	Folate	Folate	
	Iron	Iron	Iron	Iron	Iron
	Saturated fat				
Explained variation in food groups (%)	16.5	14.5	13.5	13.8	14.2
Explained variation in responses (%)	10.7	12.1	15.0	16.1	17.8
